# Imaging of the Glucose-Dependent Insulinotropic Polypeptide Receptor Using a Novel Radiolabeled Peptide Rationally Designed Based on Endogenous GIP and Synthetic Exendin-4 Sequences

**DOI:** 10.3390/ph16010061

**Published:** 2022-12-31

**Authors:** Irina Velikyan, Martin Bossart, Torsten Haack, Iina Laitinen, Sergio Estrada, Lars Johansson, Stefan Pierrou, Michael Wagner, Olof Eriksson

**Affiliations:** 1 Science for Life Laboratory, Department of Medicinal Chemistry, Uppsala University, SE-751 83 Uppsala, Sweden; 2 PET Centre, Centre for Medical Imaging, Uppsala University Hospital, SE-751 85 Uppsala, Sweden; 3 R&D Research Platform, Integrated Drug Discovery, Sanofi, 65929 Frankfurt, Germany; 4 Global Imaging, Sanofi, 65929 Frankfurt, Germany; 5 Antaros Medical AB, SE-431 53 Mölndal, Sweden

**Keywords:** GIPR, PET, insulinoma, neuroendocrine tumors

## Abstract

Imaging and radiotherapy targeting the glucose-dependent insulinotropic polypeptide receptor (GIPR) could potentially benefit the management of neuroendocrine neoplasms (NENs), complementing clinically established radiopharmaceuticals. The aim of this study was to evaluate a GIPR-targeting positron emission tomography (PET) radioligand with receptor-specific binding, fast blood clearance, and low liver background uptake. The peptide DOTA-bioconjugate, C803-GIP, was developed based on the sequence of the endogenous GIP(1-30) and synthetic exendin-4 peptides with selective amino acid mutations to combine their specificity for the GIPR and in vivo stability, respectively. The ^68^Ga-labeled bioconjugate was evaluated in vitro in terms of binding affinity, specificity, and internalization in HEK293 cells transfected with the human GIPR, GLP1, or GCG receptors and in sections of human insulinoma and NENs. In vivo binding specificity, biodistribution, and tissue background were investigated in mice bearing huGIPR-HEK293 xenografts and in a pig. Ex vivo organ distribution, pharmacokinetics, and dosimetry were studied in normal rats. [^68^Ga]Ga-C803-GIP was stable and demonstrated a high affinity to the huGIPR-HEK293 cells. Binding specificity was demonstrated in vitro in frozen sections of NENs and huGIPR-HEK293 cells. No specific uptake was observed in the negative controls of huGLP1R and huGCGR cells. A novel rationally designed PET radioligand, [^68^Ga]Ga-C803-GIP, demonstrated promising binding characteristics and specificity towards the GIPR.

## 1. Introduction

The glucose-dependent insulinotropic polypeptide receptor (GIPR) is an emerging therapeutic and diagnostic target. Its endogenous ligand is the glucose-dependent insulinotropic polypeptide (GIP), which is a 42-mer peptide released from the intestinal K-cells in response to food intake. The GIP is an incretin hormone and augments insulin secretion in response to hyperglycemia by activating the GIPR on the pancreatic beta cells. The activity of the GIPR has also been shown to affect lipid metabolism as well as bone turnover. The GIPR is expressed physiologically in low density throughout the body [1].

Overexpression of the GIPR has been demonstrated in pathologies, such as a broad spectrum of human gastrointestinal, pancreatic, and bronchial neuroendocrine neoplasms (NENs), originating from endocrine cells located in the pituitary, thyroid, pancreas, and adrenal glands or in disseminated endocrine tissues of the lung or along the intestinal tract. Importantly, a high overexpression of the GIPR occurs in SSTR-negative and GLP-1R-negative NENs [2]. This opens the possibility to use GIP-based radioligands as an alternative or complementary diagnostic and therapeutic means to the clinically established radiopharmaceuticals accumulating in the NENs via the monoamine pathway (^18^F-DOPA or ^11^C-5-HTP) or receptor binding (^68^Ga-DOTA-TOC, ^68^Ga-DOTA-TATE, and ^68^Ga-DOTA-NOC) [3,4].

The increasing realization of the potential clinical application invoked the development of GIP-based analogs for applications in imaging. A truncated analog, GIP(1-30) of the native GIP(1-42), demonstrated high potency [5], and it was used for the development of GIP-based radioligands comprising In-111 and Ga-68 for the in vivo targeting of GIPR-positive tumors [6]. The analogs demonstrated specific internalization into GIPR-transfected pancreatic endocrine cells (INR1G9-huGIPR) and specific tumor uptake in INR1G9-huGIPr mouse xenografts. However, endogenous GIP(1-30) is rapidly metabolized in vivo, and the stability of close analogs of this peptide is uncertain. Furthermore, the affinity of the abovementioned analogs was in excess of 10 nM, and no specific binding in tissues with physiological levels of GIPR expression, e.g., the pancreas, could be discerned.

To further enable quantitative and sensitive imaging of the GIPR in vivo, a novel GIP analog with sub-nanomolar affinity for the GIPR was, therefore, developed and used for assessing drug occupancy at the GIPR in the pancreas in non-human primates [7]. Their application for the measurement of receptor occupancy in the pancreas by positron emission tomography proved the high selectivity and affinity towards the GIPR.

Thus, the GIPR could constitute a target for the diagnostic scanning of NENs given the availability of the respective radioligand of high-binding affinity. Another key aspect of the development, besides the binding affinity, is the assurance that the radiopharmaceutical of interest demonstrates low liver background uptake. It is justified by the fact that the liver is one of the most common sites for the metastases of NENs [8]. Previously reported GIPR high-affinity radioligands demonstrated relatively high liver background [7], demonstrating the potential for further optimization in this aspect for potential applications in NEN diagnosis and therapy.

The radionuclide choice in this study fell on ^68^Ga. The most crucial advantage of ^68^Ga(III) is its availability from a simple ^68^Ge/^68^Ga generator system on-demand, independent of external distributors or a cyclotron. As a positron emitting radionuclide, ^68^Ga presents the advantages that PET technology offers. Its radionuclide characteristics allow for high-quality PET images, a low radiation dose to the patient and personnel, a short scanning time, and the possibility of repetitive examinations. Its chemical characteristics allow for highly reproducible and straightforward labeling chemistry amenable to automation and kit preparation [9,10,11,12]. Even though the ^68^Ga-labeling of vector molecules comprising a DOTA chelator moiety is straightforward and site specific, it requires optimization of the reaction temperature, pH, buffers, radical scavengers, and purification, taking into consideration not only the complexation reaction per se but also the properties of the vector molecules.

This study presents a rationally designed GIPR-selective analog based on the combined sequences of GIP(1-30) and exendin-4 to take advantage of the former’s specificity to the GIPR and demonstrated in vivo stability of the latter along with its preclinical characterization for in vivo localization and quantification of the GIPR-expressing NENs.

## 2. Results

### 2.1. Chemistry and Biological Assays of C803-GIP and Ga-C803-GIP

C803-GIP has a sequence partially similar to that of hGIP(1-30), wherein L-Alanine at position 2 was exchanged to D-Alanine, Methionine at position 14 was exchanged to Leucine, Aspartic acid at position 21 was exchanged to Glutamic acid, Valine at position 23 was exchanged to Isoleucine, Asparagine at position 24 was exchanged to Glutamic acid, Leucine at position 27 was exchanged to Lysine, and both Glutamine at position 29 and Lysine at position 30 were exchanged to Glycine. The sequence was amended with Histidine (31), Proline (32), and seven more residues corresponding to those found in exendin-4 (Serine(33)-Glycine(34)-Alanine(35)-Proline(36)-Proline(37)-Proline(38)-Serine(39)). The peptide sequence was conjugated via terminal Serine(39) to a DOTA chelator moiety functionalized with a vinyl sulfone linker and cysteine amide (Table 1). C803-GIP was loaded with stable isotopes of gallium to yield Ga-C803-GIP for the subsequent investigation of the stability and biological activity. Ga-C803-GIP was stable with a purity of over 95% for at least one week at ambient temperature and pH 4.5.

**Figure 1:**
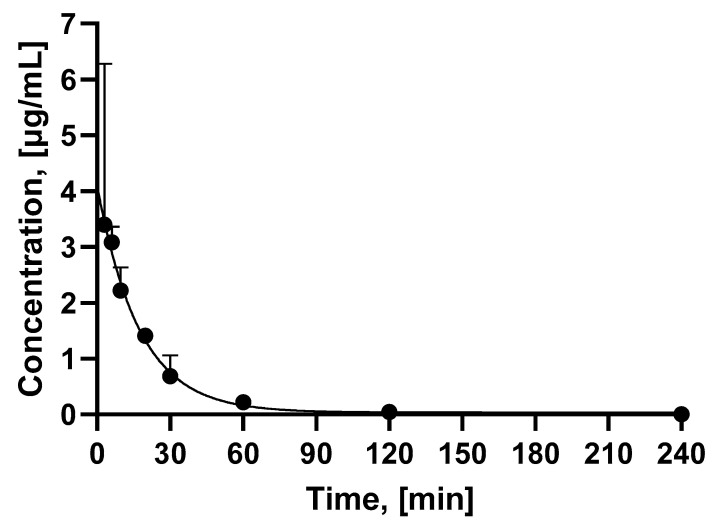
Plasma concentration of Ga-C803-GIP measured at 3, 6, 9.6, 19.8, 30, 60, 120, and 240 min post subcutaneous administration in rats (*n* = 3). The error bars are not seen due to the low SD values. The plasma concentration of Ga-C803-GIP was below the limit of detection at the 240 min time point and was assigned zero value.

The potency of the peptide conjugate in terms of EC_50_ was studied using C803-GIP and C803-GIP loaded with a stable isotope of gallium (Ga-C803-GIP) in HEK293 cells transfected with the GIPR from human, monkey (NHP), and rat species. In addition, the cross-reactivity was studied in HEK293 cells transfected with the GLP1-R and GCGR. No statistically significant differences could be found between the peptide conjugates with and without stable gallium and between the human and NHP GIPR (Table 2). In contrast, the EC_50_ for the rat GIPR was one order of magnitude poorer. The potency towards the GLP-1R and GCGR was 10,000–1,000,000-fold lower compared to that towards the GIPR. The pharmacokinetic properties were investigated in vivo in rats. Figure 1 shows the blood concentration of Ga-C803-GIP as a function of time. The subcutaneous administration of Ga-C803-GIP (1 mg/kg) showed a plasma half-life of 0.38 h. The average plasma concentration calculated to the infinity (AUC_inf_) was 1370 h*ng/mL. The plasma clearance (CL) was 0.73 L/h/kg.

### 2.2. Radiochemistry

**Figure 2:**
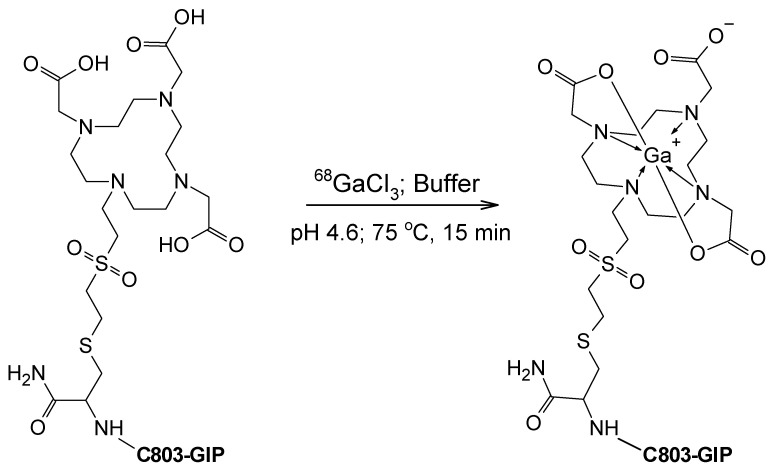
^68^Ga-labeling scheme of C803-GIP, wherein C803-GIP stands for YaEGTFISDYSIALDKIHQQEFIEWLKAGGHPSGAPPPS*.

The ^68^Ga-labeling procedure was optimized in terms of the radiochemical yield and required optimization of such factors as temperature, heating time, radical scavenger content, buffer concentration, pH, and product purification mean. The labeling was reproducible with a high non-decay corrected radiochemical yield of 61.1 ± 2.9% and a molar activity of 59.8 ± 4.4 MBq/nmol at the end of the synthesis (Figure 2). The radioactivity at the end of the synthesis was 424 ± 29 MBq with a radiochemical purity of 94.5 ± 0.3% (for the chromatogram, see Supplementary Materials, Figure S1). The respectively high molar activity allowed for the accurate determination of binding affinity and in vivo localization of the target GIPR. Although the radiolysis could be suppressed by the addition of only ethanol (200 µL) to the reaction mixture, the post-production stabilization at ambient temperature required the addition of ascorbic acid with a radiochemical purity of over 91% for at least 2 h. The accurate determination of the peptide concentration is critical for the estimation of binding affinity and was accomplished by UV-HPLC with respective response calibration.

### 2.3. Cellular Internalization and Binding Affinity of [^68^Ga]Ga-C803-GIP

**Figure 3:**
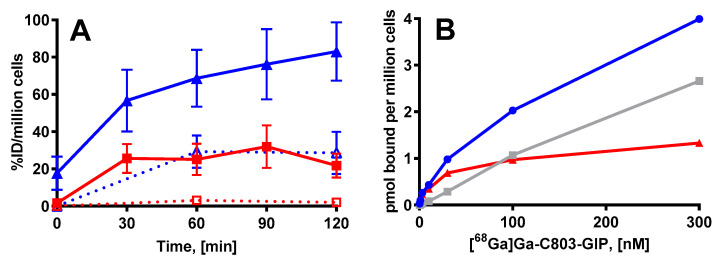
(**A**): Total binding (blue) and internalization (red) of [^68^Ga]Ga-C803-GIP in HEK293 cells expressing the GIPR presented as a function of time at 37 °C (solid lines) and at 4 °C (dotted lines) for the suppression of internalization. (**B**): GIPR binding saturation was achieved by gradually increasing the concentration of [^68^Ga]Ga-C803-GIP. The total binding (blue), non-specific binding (grey), and specific binding (red, calculated by the subtraction of non-specific binding from the total binding). Data from a representative experiment are shown.

HEK293 cells transfected with human GIPR were subjected to incubation with [^68^Ga]Ga-C803-GIP for 0, 30, 60, 90, and 120 min. The radioactivity associated with the cells (%ID/million cells) is presented as a function of time in Figure 3. The tracer bound to the cells reached approximately 75% ID/million cells after 60–120 min. The internalized fraction was approximately 40% of the total binding and could be suppressed almost completely by incubation at low temperature (4 °C). The binding of [^68^Ga]Ga-C803-GIP to huGIPR could be inhibited by 0.3 µM C803-GIP. The binding saturation assay resulted in K_d_ 18.3 ± 6.5 nM and Bmax 2.4 ± 1.0 pmol/Mcells (*n* = 3) (Figure 3B).

### 2.4. In Vitro Binding of [^68^Ga]Ga-C803-GIP to Frozen Sections and Stability in Human Plasma

**Figure 4:**
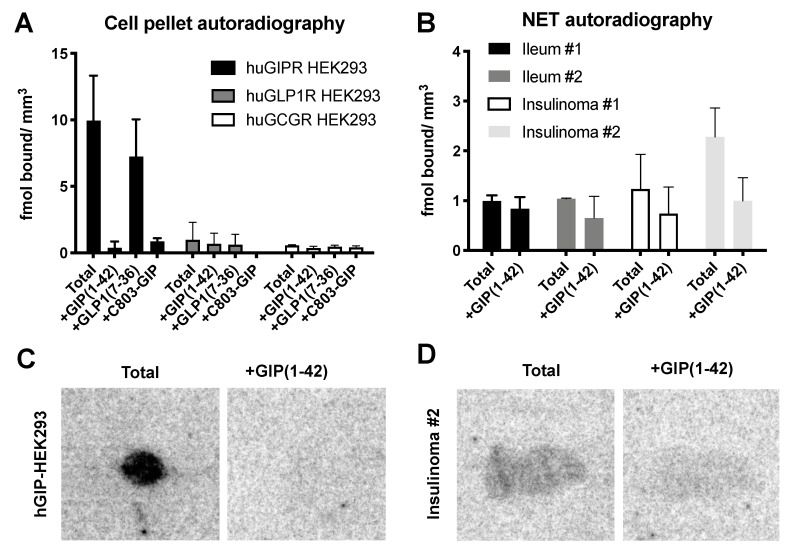
(**A**) Verification of the binding specificity of [^68^Ga]Ga-C803-GIP in huGIPR-HEK293, huGLP1R-HEK293, and hGCG-HEK293 cell pellet sections. The uptake was challenged using 10 µM GIP(1-42), GLP1(7-36), or C803-GIP in all cell lines. (**B**) Binding of [^68^Ga]Ga-C803-GIP to frozen sections of human biopsies of NETs from ileum and insulinomas of the pancreas in the presence and absence of 5 µM GIP(1-42). (**C**,**D**) Representative autoradiograms of [^68^Ga]Ga-C803-GIP binding to frozen sections of hGIP-HEK293 cell pellets and insulinoma #2.

The binding of [^68^Ga]Ga-C803-GIP to sections of a frozen pellet of cells transfected with the GIPR was strong and could be abolished by co-incubation with 10 µM GIP(1-42) and 10 µM C803-GIP but not by GLP1(7-36) (Figure 4). No uptake of [^68^Ga]Ga-C803-GIP and no blocking effect could be detected in the pellets of cells transfected with the hGLP1R and hGCGR. One set of frozen sections of biopsies of human INS and ileum NETs was subjected to the incubation with [^68^Ga]Ga-C803-GIP for the elucidation of total binding. Sections of the hGIPR-HEK293 cell pellet were included as positive controls and hGLP1R-HEK293 cell pellet sections as negative controls. The other set of the sections was pre-incubated with GIP(1-42) prior to the addition of [^68^Ga]Ga-C803-GIP in order to elucidate the binding specificity. A slight blocking effect could be achieved in the frozen sections of human INS but not ileum NETs.

[^68^Ga]Ga-GIP-C803 was stable in human plasma for at least 90 min (See Supplementary Materials, Figure S2).

### 2.5. In Vivo Organ Distribution of [^68^Ga]Ga-C803-GIP in Rat

**Figure 5:**
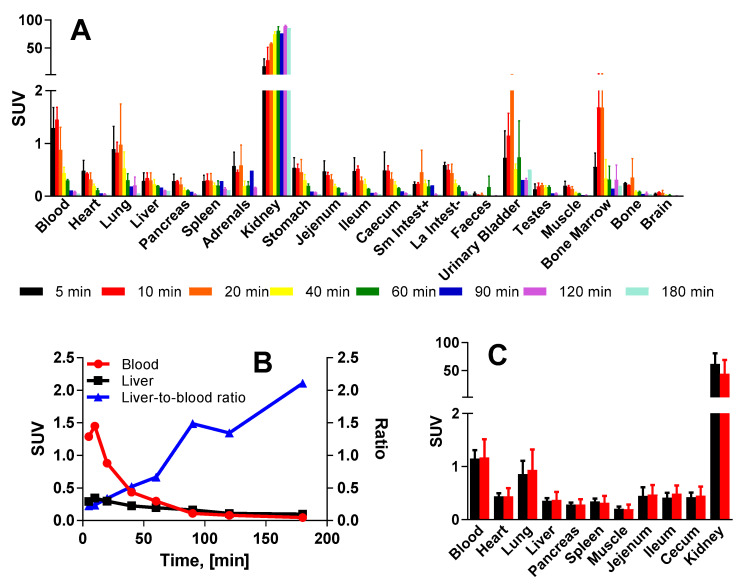
(**A**) Organ distribution of [^68^Ga]Ga-C-803-GIP in male rats. Most tissues except kidney demonstrated rapid washout. (**B**) Radioactivity clearance from blood (red) and liver (black), and liver-to-blood ratio as a function of time (blue curve). (**C**) Organ distribution of [^68^Ga]Ga-C803-GIP in rat showing lack of blocking effect when comparing the base-line study (only [^68^Ga]Ga-C-803-GIP; *n* = 4; black bars) and the competition study ([^68^Ga]Ga-C-803-GIP and excess of non-labeled precursor, C803-GIP; *n* = 4; red bars).

The organ distribution kinetics of [^68^Ga]Ga-C803-GIP was studied in male Sprague Dawley rats (*n* = 16). Two animals were sacrificed at each of the eight time points as depicted in Figure 5A. Most tissues exhibited a rapid clearance of radioactive uptake except for the kidney, which is an excretory organ. The liver-to-blood ratio increased with time indicating faster blood clearance compared to the washout from the organ (Figure 5B).

Four rats received a co-injection of 1 mg/kg of C803-GIP precursor in the competition experiment. The in vivo competition showed no significant displacement in the liver or pancreas or in any other organ in the presence of an excess of non-labeled precursor C803-GIP at 20 min post injection (Figure 5C).

### 2.6. Organ Distribution of [^68^Ga]Ga-C803-GIP in GIPR-Expressing Xenograft Mice

**Figure 6:**
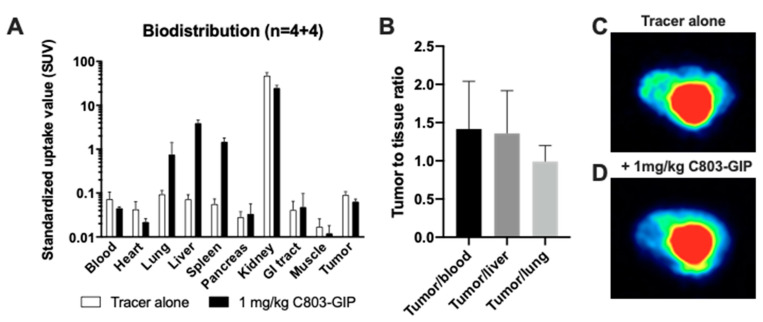
(**A**) In vivo biodistribution in mice carrying a GIPR-expressing xenograft tumor. (**B**) Tumor-to-tissue background ratios for some tissues with potential NEN metastasis. Representative PET images (transaxial projections) showing the binding of [^68^Ga]Ga-C803-GIP in xenograft tumor alone (**C**) or challenged by unlabeled C803-GIP (**D**). White arrows indicate the location of the tumor, and red arrows indicate the bladder.

The binding to the GIPR-expressing xenografts was low (Figure 6A,C). The expression of the GIPR in the inoculated cell line was unexpectedly decreased after in vivo transplantation but still present as determined by an ex vivo autoradiography assay (in the range of 3 fmol/mm^3^). The tumor-to-tissue ratio was in the range of 1.5 for blood and liver (Figure 6B). Furthermore, there was only a limited blocking effect (on average, approximately a 30% decrease) seen by co-injection of 1 mg/kg C803-GIP (Figure 6A,D).

### 2.7. In Vivo Organ Distribution of [^68^Ga]Ga-C803-GIP in Piglets

**Figure 7:**
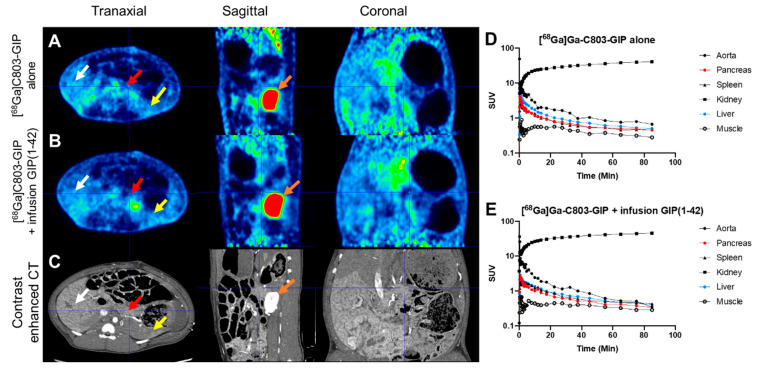
PET/CT images of the abdomen of pig following administration of [^68^Ga]Ga-C803-GIP, either tracer alone (**A**) or during infusion of GIP(1-42) (**B**). Anatomical reference by contrast enhanced CT (**C**). White arrows indicate liver, red arrows indicate pancreas, orange arrows indicate kidney, and yellow arrows indicate spleen. Images are normalized to SUV = 2 and are summations 60–90 min after tracer administration. Time-dependent kinetics in relevant tissues are presented after administration of [^68^Ga]Ga-C803-GIP alone (**D**) or during intravenous infusion of GIP(1-42) (**E**).

[^68^Ga]Ga-C803-GIP demonstrated a rapid clearance from the circulation and most tissues after administration as assessed by dynamic PET/CT (Figure 7). Uptake in the liver was SUV <1 after 30 min and was approximately SUV ≈ 0.5 after 90 min, indicating low non-specific accumulation due to metabolism. Excretion occurred almost exclusively via the urine as demonstrated by the strong and irreversible retention in the kidney cortex. Binding in the pancreas was low and similar in magnitude to that in blood and the spleen. Furthermore, pre-administration of GIP(1-42) did not reduce the uptake of [^68^Ga]Ga-C803-GIP in any tissue, indicating a limited blocking of the tracer bound specifically to the GIPR (Figure 7, for the whole-body image see Supplementary Materials, Figure S3).

### 2.8. Human Predicted Dosimetry

The rat organ distribution of [^68^Ga]Ga-C803-GIP data were extrapolated to human tissues and used for the calculation of the predicted absorbed radiation dose in humans (Table 3). The doses were highest in the kidneys for both males (0.713 mGy/MBq) and females (0.773 mGy/MBq). The whole-body effective dose was 0.0217 mSv/MBq for males and 0.0237 mSv/MBq for females.

## 3. Discussion

Most NENs express the GIPR, GLP-1R, or/and somatostatin receptor (SSTR) [13]. Targeting the SSTR for SPECT and PET imaging as well as radiotherapy is an established method for the management of NENs [14,15,16]. SPECT and PET imaging in combination with exendin-based radiopharmaceuticals have also been proven clinically relevant [17,18,19]. The expression of the GIPR, and thus the potential for targeting imaging, has been demonstrated for pancreatic and gastrointestinal NENs [20,21], and the development of radioligands for specific receptor binding has been initiated [6,22]. The endogenous GIP(1-42) was truncated to GIP(1-30) and functionalized with a DOTA chelator for the subsequent labeling with In-111 and Ga-68 (EG2, EG4) [6], and the mouse xenografts (INR1G9-huGIPr) were visualized, though the affinity of the radioligand was reduced compared to GIP(1-42). In an attempt to maintain the binding affinity, the endogenous GIP(1-42) was functionalized at Lys37 with a DTPA chelator ([Lys37(111In-DTPA)]N-acetyl-GIP1-42), and the resulting radioligand visualized the mouse xenografts (BHK-GIPR) [22]. Another GIP analog, consisting of the truncated part of GIP(1-31) with a change of 13 amino acid residues at various positions essential for binding and stability, was labeled with Gallium-68 ([^68^Ga]Ga-S02-GIP-T4) and demonstrated sub-nanomolar affinity for the GIPR [7]. Importantly, cross-reactivity for the GLP-1R was negligible, indicating high selectivity of the radioligand for the GIPR. The sensitivity of the imaging was high allowing for the first-time visualization of the GIPR density in the physiological pancreas, most probably due to a magnitude higher affinity compared to previously published analogs. However, while [^68^Ga]Ga-S02-GIP-T4 enables the sensitive detection of the pancreatic GIPR, it demonstrated a liver background signal of approximately SUV 8 in non-human primates after 90 min, potentially making detection of the GIPR in this tissue challenging, e.g., in the form of NEN metastasis. Given the knowledge of the liver being one of the most common sites for metastases [8], there is still room for novel GIPR-targeting radioligands with a specific focus on the detection of primary and metastatic NENs and low liver background uptake.

The C803-GIP reported in this work is based on the GIP(1-30) and exendin-4 sequences. The aim of the design was a peptide of improved water solubility, in vivo stability, and binding-site recognition. Eight of the amino acid residues of GIP(1-30) at positions 2, 14, 21, 23, 24, 27, 29, and 30 were substituted with other AAs unique to C803-GIP. The exchange of *L*-Ala(2) to *D*-Ala(2) was aimed to improve the stability against enzymatic processing. In order to exclude the oxidative radiolysis, Met(14) was substituted with Leu(14). Substitution of aspartic acid with glutamic acid is known to increase the unfolding transition temperature of a protein [23]; thus, the introduction of Glu(21) instead of Asp(21) was considered. Substitution of Val(23) to Ile(23) would introduce more bulkiness adjacent to the protein backbone and restrict the conformation to β-sheets. The exchanges of Asn (24) to Glu(24) and Leu(27) to Lys(27) were to improve solubility. Both Gln(29) and Lys(30) were substituted with Gly residues to support the tryptophan cage conformation. The C-terminal tail (31-39), originating from exendin-4, provided stability and solubility [24], while no interaction with the receptor was proven by the absence of cross-activation in the affinity studies (Table 2). Pro(31) was exchanged to His(31) in order to reduce the constraint of the structure and add a positive charge for better binding functionality.

In comparison to the previously described S02-GIP-T4 analog, the potency of C803-GIP was seemingly improved with regard to the human and NHP GIPR (Table 2). This effect may be due to the exchange of Aib(2) to A(2), L(10) to Y(10), R(16) to K(16), E(20) to Q(20), L(27) to K(27), K(31) to H(31), and the addition of the C-terminal tail ((32-39); PSGAPPPS). [^68^Ga]Ga-C803-GIP was furthermore evaluated for its affinity, internalization, and binding specificity towards the GIPR. Despite the improved potency, the affinity of [^68^Ga]Ga-C803-GIP (K_d_ of 18.3 ± 6.5 nM and Bmax approximately 2.4 ± 1.0 pmol/Mcells (*n* = 3) was twenty-times poorer than that of [^68^Ga]Ga-S02-GIP-T4 (K_d_ of 0.87 ± 0.11 nM) [7]. The affinity of [^68^Ga]Ga-C803-GIP was comparable in magnitude to that of EG2 and EG4 (Kd values of 10.6 ± 1.6 nM for 111/natIn-EG2 and 8.5 ± 1.0 nM for 111/natIn-EG4) [6] and higher than that of [Lys37(111In-DTPA)]N-acetyl-GIP1-42 (IC_50_ = 4.8 µM) [22]. The notion according to the binding potential (B_max_/K_d_) on the linear improvement of imaging of a certain receptor density (B_max_) with the improvement of radioligand affinity (K_d_) is reflected in these results.

The binding specificity of [^68^Ga]Ga-C803-GIP was investigated in vitro in cell and frozen tissue sections as well as in vivo in mice bearing xenografts. Several sets of frozen sections of HEK293 cell pellets were used for the investigation of the binding specificity: one transfected with huGIPR and the other with huGCGR or huGLP1R. The experiment outline also allowed the investigation of the cross-activity. In both cases, the blocking of the total uptake of [^68^Ga]Ga-C803-GIP was conducted using an excess of GIP(1-42), C803-GIP, or GLP(7-36). The blocking effect of native GIP(1-42) and non-labeled precursor C803-GIP was strong, respectively by 99 and 92%. There was no baseline accumulation or blocking effect observed in the huGLP1R-HEK293 or huGCGR-HEK293 cell pellet sections, demonstrating the specificity of [^68^Ga]Ga-C803-GIP binding towards the GIPR and absence of cross-reactivity towards the GLP1R and GCGR. A similar tendency was observed in the frozen sections of the human biopsies of the INS and ileum of pancreatic NET, wherein the inhibition of the [^68^Ga]Ga-C803-GIP binding by GIP(1-42) was detected. Thus, in summary, the in vitro data demonstrated strong GIPR-mediated binding to cell lines and relevant tumor biopsies supporting further in vivo evaluation.

[^68^Ga]Ga-C803-GIP uptake, background binding, and washout were investigated in healthy rats to ensure low physiological uptake in the background tissues necessary for providing sufficiently high-contrast images with lesion-elevated uptake. The distribution indicated a fast clearance from all tissues except for the kidneys as the organ of excretion. The organ most commonly susceptible to metastases is the liver [8], and thus a low physiological uptake is essential for achieving an image contrast. The liver uptake was below SUV <1 already after 5 min post-administration, and the washout with respect to the 5 min time point was 50% within 1 h and 75% within 3 h, promising favorable imaging conditions to detect GIPR-expressing hepatic lesions. [^68^Ga]Ga-C803-GIP also displayed a rapid clearance from blood. Essentially, the only organ displaying a high SUV increasing with time up to 85.5 ± 1.6 within 3 h was the kidney indicating renal excretion. Thus, no uptake significantly higher than the background signal could be detected for the rest of the vital organs. Moreover, no statistically significant difference could be found in the organ distribution between the baseline, wherein only the radioligand was administered, and the competition study, wherein an excess of C803-GIP was co-administered with the radioligand. The low physiological uptake and absence of the blocking effect indicate a non-existent expression of the GIPR and thus strong potential for high contrast of the images with NENs. In particular, the ratio of SUVs for liver-to-blood increased with the time, which is important given that the liver is a usual organ for metastases. However, the liver-to-blood ratio is driven primarily by the rapid clearance of [^68^Ga]Ga-C803-GIP from blood, and there may be an increased uncertainty for this value as the liver uptake is divided by a very low blood SUV value. Although the affinity of [^68^Ga]Ga-C803-GIP was comparable or somewhat lower to the GIPR targeting analogs previously reported [6,7,22], the liver uptake was lower for [^68^Ga]Ga-C803-GIP presenting a crucial advantage for detection of hepatic lesions.

The in vivo binding specificity of [^68^Ga]Ga-C803-GIP was lastly studied in vivo in mice bearing xenografts of GIPRhu HEK293 cells, wherein the uptake in the xenografts was partly precluded by the excess of C803-GIP as compared to the baseline uptake. The uptake in the muscle, liver, and lung was low and non-specific potentially allowing images of high contrast in these tissues, which are potential locations for NEN metastasis. However, the tumor uptake in absolute terms was very low (SUV < 1), which is hardly suitable for sensitive in vivo detection of GIPR-expressing neoplasms. Furthermore, the tumor-to-tissue ratio was just above 1.

A similar biodistribution pattern was seen by PET/CT scanning in pig. A very low background signal was seen in most tissues. The liver uptake was SUV ≈ 0.5 after 90 min, which was approximately 16-times lower than for a previously reported GIPR high-affinity PET tracer in a large animal model [7]. However, the binding of [^68^Ga]Ga-C803-GIP to tissues with a known distribution of the GIPR such as the pancreas was low or negligible. Furthermore, the blocking of the GIPR by an infusion of GIP(1-42) did not affect the uptake pattern in pig.

The radiotoxicity was investigated in order to determine the possibility of repeated examinations for follow-up and longitudinal studies. The organ distribution of [^68^Ga]Ga-C803-GIP in healthy rats was thus analyzed for the extrapolation to human radiation dosimetry. The calculation of the organ-equivalent doses and effective dose was based on the assumptions that the biodistribution pattern is similar in a human and rat, and that radioactivity is distributed homogeneously throughout a given organ. The longest incubation time of 180 min corresponded to over three effective half-lives covering both the physical decay and biological clearance, and thus providing high accuracy for dosimetry calculations. The kidney was found to be the critical organ tissue in the human predicted dosimetry as it was for [^68^Ga]Ga-S02-GIP-T4 calculated based on rat biodistribution data; however, the absorbed dose was 1.5-times higher for the former. It was somewhat higher also for adrenals and the spleen (app. 1.2 times), while the absorbed dose of [^68^Ga]Ga-S02-GIP-T4 was considerably higher in the small intestine (2.2 times), heart wall (2.4 times), liver (1.5 times), lung (1.3 times), red marrow (2 times), osteogenic cells (1.9 times), ovaries (1.5 times), thymus (2 times), thyroid (1.7 times), and urinary bladder wall (2.5 times) as compared to the [^68^Ga]Ga-C-803-GIP absorbed dose to those organs. Although the absorbed dose was higher for [^68^Ga]Ga-S02-GIP-T4 in most of the organs, the total effective dose was still somewhat lower. Importantly, both [^68^Ga]Ga-C-803-GIP and [^68^Ga]Ga-S02-GIP-T4 would potentially allow for several PET scans per year with regard to the total effective dose. Renal dosing should ideally be decreased to enable additional PET scanning. Additionally, renal dosimetry is crucial for future potential development of radiotherapeutic applications of the current compound. Kidney uptake may potentially be decreased by the pre-administration of amino acids or plasma volume expanders, similar to how these strategies are employed for established Peptide Receptor Radiotherapies. We also hypothesize that renal uptake can be modulated by altering the rapid clearance of [^68^Ga]Ga-C803-GIP by adding a fatty acid moiety or albumin-binding domain. A long circulatory half-life may simultaneously increase the binding capacity and tissue exposure of [^68^Ga]Ga-C803-GIP.

Thus, [^68^Ga]Ga-C803-GIP demonstrated strong and GIPR-specific binding in vitro in cells and tissues. Of importance, we demonstrated GIPR-mediated binding in sections from human insulinoma and ileum NENs. The in vivo tissue background and excretion patterns were favorable for potential tumor and liver metastases localization, but binding to the GIPR-dense tissues was low. The reason for poor translation of the promising in vitro data to the in vivo setting may have several reasons, but the primary culprits are likely the limited affinity (15–20 nM) towards the GIPR, perhaps in combination with low tissue exposure due to rapid clearance. However, the advantage of [^68^Ga]Ga-C-803-GIP was the in vivo low liver uptake, potentially allowing high-contrast imaging of liver metastatic lesions. Thus, further development of [^68^Ga]Ga-C-803-GIP as a lead compound is warranted with the major aims of developing a radiotracer with improved binding to GIPR-rich tissues in combination with a reduced rate of clearance and improved biodistribution while preserving low liver uptake.

## 4. Methods and Materials

### 4.1. Generation of Sequence and DOTA Conjugation

The peptide conjugate C803-GIP was developed based on the hGIP(1-30) and exendin-4 sequences (Table 1). Selective amino acid mutations were applied to attain selectivity for the GIPR and improved stability. As seen in Table 1, C803-GIP has 10 residues shared with both GIP(1-30) and exendin-4. Twelve residues, and especially residues 12–20, are conserved from GIP(1-30) to retain the strong affinity and potency towards the GIPR. Finally, 10 residues towards the C-terminus are conserved from exendin-4 to take advantage of the established high in vivo stability of this peptide. The alanine was changed to d-alanine in order to minimize enzymatic degradation at the N-terminal. The peptide conjugate comprises the chelating moiety DO3A attached via a cysteine amide on the C-terminus and a diethylsulfone linker. The peptide was synthesized in-house via standard solid-phase peptide synthesis (Sanofi). LC-ESI-TOF-MS analysis of C803-GIP was performed using an LCT Premier mass analyzer. The analysis was performed with positive-mode scanning and selected ion recording, detecting [M+6H]^6+^, [M+5H]^5+^, [M+4H]^4+^, [M+3H]^3+^, and [M+2H]^2+^ ions of C803-GIP. Reconstitution of the data resulted in a monoisotopic mass of 4822.3 Da (see Supplementary material, Figure S4).

### 4.2. In Vitro Potency

The potency of C803-GIP loaded with nonradioactive gallium was assessed by a functional cAMP assay in the HEK293 cells transfected with the huGIPR (EvoTech). The details of the cell line and potency assay procedure have been described previously [25,26].

### 4.3. Radiolabeling of C803-GIP

The ^68^Ge/^68^Ga generator (pharmaceutical grade, GalliaPharm^®^, Eckert & Ziegler, Berlin, Germany) eluate was fractionated, and the top fraction of 3.5 mL containing approximately 90% of the radioactivity was collected for the radiolabeling. The solution was buffered with sodium acetate buffer (1 M, 300 µL, pH 6–7) to achieve a pH of 4.6–5.0. To suppress the radiolysis, ethanol (200 µL) was added to the reaction mixture, and ascorbic acid (8–10 mg) was added to the final product. The peptide conjugate C803-GIP (20–40 nanomole) dissolved in acetate buffer (pH 6–7) was added to the buffered ^68^Ga-eluate solution, and the reaction mixture was heated at 75 °C for 15 min. The reaction mixture was purified using a solid-phase extraction cartridge (HLB, Oasis) to assure the elimination of possible hydrophilic radioactive impurities, germanium-68, and colloids. The product was eluted with 1 mL of 60% ethanol in phosphate buffer solution. The radiochemical purity and the peptide concentration in the product were determined by high-pressure liquid chromatography (HPLC). The HPLC system (LaChrom, Hitachi, VWR, Atlanta, GA, USA) consisted of an L-2130 pump, UV detector (L-2400), and a radiation flow detector (Bioscan, Wheaton, IL, USA) coupled in series for product quality control. Separation of the analytes was accomplished using an end-capped analytical column with stationary reversed phase (C-4; Vaydac-C4; 50 × 4.6 mm; particle size: 3 µm). The following system was used: A = 10 mM TFA; B = 30% water: 70% acetonitrile: 10 mM TFA with UV-detection at 220 nm; linear gradient elution: 2–9.9 min from 20% to 100% B followed by re-equilibration 9.9–10 min from 100% to 20% B; flow rate was 1.0 mL/min. Data acquisition and handling were performed using the EZChrom Elite Software Package. The stability of the product at room temperature was monitored for 2 h and assessed by UV-Radio-HPLC. The final product was formulated dependent on the biological assay. The total radioactivity of the product was then measured in an ionization chamber. The radioactivity recovery from the analytical column was controlled by performing an analysis of the product, generator eluate, and the product spiked with the generator eluate with and without column. The fractions were collected from the outlet of the HPLC system for the subsequent measurement of the radioactivity in a well-type NaI(Tl) scintillation counter corrected for dead-time and for radioactive decay. The recovery was calculated as a fraction of the radioactivity collected from the run with the column in relation to the radioactivity collected from the run without the column. The recovery of the product and generator eluate was, respectively, over 98% and nearly 100%, indicating high reliability of the analysis.

### 4.4. Cellular Internalization and Binding Affinity of [^68^Ga]Ga-C803-GIP

The cell internalization assay was performed as described in detail previously [25]. Briefly, HEK293 cells transfected with the human GIPR (approximately 300,000 cells) were incubated with 5 nM [^68^Ga]Ga-C803-GIP in complete media for 0, 30, 60, 90, and 120 min, either at 37 °C or at 4 °C. To measure the membrane-bound and internalized radioligand, the cells were first treated with 0.2 M glycine buffer containing 4 M urea (pH 2.5) (acid wash buffer) for 5 min, and the supernatant (containing the membrane-bound fraction) was measured in a gamma counter. Thereafter, the cells were treated with 1 M NaOH (basic wash buffer) for 30 min, and the lysed cells were measured by a gamma counter (internalized fraction). The assay was repeated three times. The total and internalized fractions of [^68^Ga]Ga-C803-GIP at 37 °C or at 4 °C were calculated.

For the affinity assessment, the GIPR-transfected HEK293 cells (0.5–0.6 million cells per dish) were incubated with seven concentrations (0.3, 1.0, 3.0, 10, 30, 100, and 300 nM) of [^68^Ga]Ga-C803-GIP G in 1 mL complete media for 60 min. The cells were incubated with radiotracer in the presence and absence of 10 µM of human GIP(1-42) (to block the GIPR and assess non-specific binding). The assay was performed at 4 °C to suppress internalization. After incubation, the cells were washed two times with complete media. For each dish, the cells were then trypsinized, resuspended, and counted, and their radioactivity was measured in a gamma counter. The well counter data were decay corrected to the start of each experiment and converted to pmol bound tracer per million cells (pmol/M cells). Specific binding was determined by subtracting the non-specific binding from the total binding. All samples were in triplicate, and each experiment was repeated at least three times with different batches of radioligand. The values for K_d_ and B_max_ were calculated using non-linear curve fitting in GraphPad Prism 6.05 (GraphPad, La Jolla, CA, USA).

### 4.5. Assessment of Binding Specificity In Vitro by Autoradiography

The binding specificity was assessed by an in vitro autoradiography binding assay using frozen sections of cell pellets of huGIPR-HEK293, huGCGR-HEK293, or huGLP1R-HEK293 and frozen sections of human biopsy samples of gastroenteropancreatic neuroendocrine tumor and insulinoma tumor. Biopsies from surgically removed tumors were obtained from Uppsala Biobank (sample collection 827), and their use was approved by the Swedish Ethical Review Authority (Dnr 2020-00049).

For the competition studies, human GIP(1-42), C803-GIP, or human GLP1(7-36) ligands were used, wherein the latter served as a negative control. [^68^Ga]Ga-C803-GIP at a concentration of 5 nM was incubated with frozen sections of pellets of huGIPR-HEK293, huGCGR-HEK293, or huGLP1R-HEK293 cells as described previously [27]. Another set of the sections was co-incubated with an excess of blocking ligands: 5 µM GIP(1-42), 1 µM GLP1(7-36), or 1 µM unlabeled C803-GIP. The sections were incubated at room temperature (RT) for 60 min in phosphate-buffered saline at pH 7.4 (PBS) containing 1% Bovine Serum Albumin (BSA). Next, the sections were washed 1 × 1 min in assay buffer and 2 × 1 min in PBS followed by air drying at 37 °C. The sections were then exposed to a phosphor-imager screen overnight and digitalized using a Cyclone Phosphor Imager system (PerkinElmer). Autoradiogram images were visualized and analyzed with ImageJ (NIH, US).

Frozen tissue sections (10 µm) from human insulinoma and pancreatic tumors were incubated with 5–10 nM [^68^Ga]Ga-C803-GIP in PBS containing 1% BSA for 60 min at RT. The sections were incubated with radiotracer alone or together with 5 µM GIP(1-42) or 1µM unlabeled C803-GIP to assess the binding specificity. A set of frozen sections were incubated with the tracer in the presence of 1 µM GLP1(7-36) to evaluate potential cross-binding of the radiotracer to the GLP1R. Duplicate sections were used, and each experiment was repeated at least three times with different batches of radioligand. After incubation, the sections were washed three times in PBS, carefully dried at 37 °C, and thereafter exposed to a digital phosphorimager plate overnight together with a 10 µL droplet of radioactive reference (cross-calibrated against a gamma counter) on absorbent paper attached to an object glass. The phosphor imager plates were scanned using a Cyclone Plus Phosphor imager (Perkin Elmer) at 600 dpi, and the resulting autoradiograms were analyzed by ImageJ. The pixel values in counts/mm^2^ were converted to Bq/mm^2^ by the included reference. Bq/mm^2^ was further converted to fmol/mm^2^ by the known molar activity (Bq/fmol) of [^68^Ga]Ga-C803-GIP.

### 4.6. Organ Distribution of [^68^Ga]Ga-C803-GIP in Rat

All animal experiments in this study were authorized by the Animal Ethics Committee of the Swedish Animal Welfare Agency and carried out according to the ARRIVE and institutional guidelines (“Uppsala university guidelines on animal experimentation”, UFV 2007/724).

The animals were kept at a constant temperature (20 °C) and humidity (50%) in a 12 h light–dark cycle. Food and water were provided ad libitum.

The in vivo organ distribution of [^68^Ga]Ga-C803-GIP was assessed in Sprague Dawley rats (324 ± 36 g, male, *n* = 16). The radiotracer was administered into the tail vein of unsedated animals as a bolus in 0.5–0.6 mL of PBS as the vehicle. The animals received 4.1 ± 2.2 MBq/kg ^68^Ga]Ga-C803-GIP corresponding to 2.4 ± 1.3 µg/kg peptide mass.

The in vivo organ distribution was conducted for 5 to 180 min, euthanizing two animals at each of eight time points (5, 10, 20, 40, 60, 90, 120, or 180 min). Samples of blood, heart, lung, liver, pancreas, spleen, adrenal, kidney, small intestine (without/with its content), large intestine (without its content), feces, urinary bladder (rinsed), testis, muscle, bone, bone marrow, thyroid, and brain were immediately harvested, weighed, and measured for radioactivity (gamma counter). The organ uptake of [^68^Ga]Ga-C803-GIP was corrected for radioactivity decay to the time point of administration and expressed as standardized uptake values (SUVs, Equation (1)).

(1) SUV (11)=Radioactivitytissue (Bq)Weighttissue(g)Radioactivityinjected (Bq)Weightbody(g)

The competition experiments were designed and guided by the results of the organ distribution study. Based on the observed rapid clearance of [^68^Ga]Ga-C803-GIP from the pancreas (known to contain the GIPR), 20 min post-administration was the time point selected for the blocking studies. The rats (351 ± 23 g, male, *n* = 8) were administered [^68^Ga]Ga-C803-GIP (18.4 ± 3.7 MBq/kg; 10.7 ± 2.2 µg/kg). A high radioactive dose was selected to potentially increase the tissue signal. Half of them (*n* = 4) received [^68^Ga]Ga-C803-GIP alone, while the other half was co-injected with 1 mg/kg of non-labeled C803-GIP. The animals were sacrificed 20 min after the administration of [^68^Ga]Ga-C803-GIP, and the tissue uptake of the tracer was analyzed as described above.

### 4.7. Organ Distribution [^68^Ga]Ga-C803-GIP in GIPR-Expressing Xenograft Mice

BALB/c nu/nu mice (*n* = 12, 18 ± 1 g) were inoculated with huGIPR-HEK293 cells (3 million) in the right hind leg and examined with [^68^Ga]Ga-C803-GIP after two weeks. Eight mice were used for the ex vivo organ distribution experiment, wherein one group of mice (*n* = 4) was injected in the tail vein with 0.5 MBq [^68^Ga]Ga-C803-GIP (corresponding to 4 µg/kg), and the second group of mice was injected in the tail vein with 0.5 MBq [^68^Ga]Ga-C803-GIP (corresponding to 4 µg/kg) spiked with 1 mg/kg C803-GIP. After 60 min, the mice were euthanized, and the xenograft and selected organs were excised, weighed, and measured for radioactivity. The organ uptake was expressed as SUVs.

Four additional mice were used for in vivo PET imaging, wherein one set of mice (*n* = 2) was injected in the tail vein with a target dose of 1 MBq [^68^Ga]Ga-C803-GIP alone, and the second group of mice was injected in the tail vein with 1 MBq [^68^Ga]Ga-C803-GIP spiked with 1 mg/kg C803-GIP. The injections were given to anesthetized animals who were positioned on the PET scanner bed. The dynamic PET was initiated at the same time as the tracer was administered and lasted for 60 min. Thereafter, the mice were euthanized, the organs were excised, weighed, and measured for radioactivity, and the organ uptake was expressed as SUVs. PET images were reconstructed and summed over 60 min. PET image analysis and generation of Maximum Intensity Projections (MIPS) were performed in PMOD (PMOD Technologies, Zürich, Switzerland). Coronal projection images were taken over the plane of the tumors.

### 4.8. In Vivo Organ Distribution of [^68^Ga]Ga-C803-GIP in Piglets

An anesthetized pig (25 kg) was positioned to include the abdomen with pancreas in the center of the 25 cm axial field of view of a Discovery MI PET/CT scanner (GE Healthcare, MI, USA) by assistance of a low-dose CT scout view (140 kV, 10 mAs). The details of the animal handling, preparation, and anesthesia have been previously described [28].

Attenuation correction was acquired by a 140 kV, 10–80 mA CT examination. The administration of [^68^Ga]Ga-C803-GIP (17.3 MBq, corresponding to approximately 2.5 μg peptide or 0.10 μg/kg) was made upon the start of the baseline dynamic scan lasting for 90 min (30 frames; 12 × 10 s, 6 × 30 s, 5 × 120 s, 5 × 300 s, 2 × 600 s). The dynamic scan was followed by a static scan over the brain from 90–120 min post administration. A second PET investigation was performed after an additional 2 h to allow the radioactivity from the first scan to fade from the circulation by decay and excretion.

Before the second PET examination, an infusion with human GIP(1-42) (20 pmol/kg/min) was conducted for a total of 150 min. Thirty minutes after the start of the GIP(1-42) infusion, the pig was administered with [^68^Ga]Ga-C803-GIP (20.6 MBq, corresponding to 0.13 μg peptide/kg) and scanned according to the same protocol as for the baseline scanning. The GIP(1-42) infusion was continued until the end of the PET scan. Finally, after the last PET scan, a contrast-enhanced CT scan was performed to assist delineation of the tissues, e.g., the pancreas.

Segmentation of the tissues of interest (descending aorta, pancreas, spleen, kidney, liver, and muscle) was performed in PMOD (PMOD Technologies), and time–activity curves were expressed as SUV as detailed above.

### 4.9. Stability of [^68^Ga]Ga-GIP-C803 in Human Plasma

Stability of [^68^Ga]Ga-GIP-C803 was studied in human plasma, wherein the agent was incubated at 37 °C for under 30 s, 5 min, 45 min, and 90 min. Thereafter, the samples were analyzed by polyacrylamide gel electrophoresis. To enable the interpretation of the results, a number of references were applied along with the molecular weight marker (Figure S2). The gel was imaged by GelDoc Go Imaging System (Biorad, Hercules, CA, USA) for protein/peptide signals and, thereafter, exposed to a phosphor-imager screen overnight and digitalized using the Cyclone Phosphor Imager system (PerkinElmer) for radioactivity signals. The amount of the sample radioactivity loaded on the gel was controlled by a reference drop of the same sample volume on a filter paper exposed to the same screen. The images were analyzed by ImageJ (NIH, Bethesda, MD, USA) and Image Lab (Biorad) software.

### 4.10. Human Predicted Dosimetry

The predicted dosimetry in humans was calculated using [^68^Ga]Ga-C803-GIP organ distribution data obtained in healthy male Sprague Dawley rats. The rat decay-uncorrected SUVs (SUV_A_) in various organs were normalized to the whole-body adult reference phantom weights (Equation (2)) [29] in order to assess the residence times (MBq-h/MBq) of the tracer in those organs [30]. The data from the measured time points (5, 10, 20, 40, 60, 90, 120, and 180 min) were used for trapezoidal approximation followed by the extrapolation from the last point to infinity by a single exponential fit. The bone marrow blood volume model was used for the determination of the residence time in bone marrow. The absorbed doses were calculated using OLINDA/EXM version 1.1 with reference to adult male and female phantoms (International Commission on Radiological Protection 60).

(2) $${\left[\frac{\%}{\mathrm{organ}}\right]}_{\mathrm{human}}={\mathrm{SUV}}_{A}\times {\left(\frac{g_{\mathrm{organ}}}{{\mathrm{kg}}_{\mathrm{weight}}}\right)}_{\mathrm{human}}$$

### 4.11. Statistics

The data on group level are reported as means ± SD. The differences between the groups were assessed by one-way ANOVA.

## 5. Conclusions

A novel peptide construct, C803-GIP, was strategically designed based on the sequences of GIP(1-30) and exendin-4. The radiolabeled analog [^68^Ga]Ga-C803-GIP demonstrated potential for the visualization of NENs having in vitro binding and selectivity towards the GIPR. However, in vivo binding of [^68^Ga]Ga-C803-GIP in GIPR-expressing tissues, including xenografts and the pancreas, was relatively low, indicating limited potential in diagnostics. However, in vivo liver uptake was low, potentially providing high-contrast localization of liver metastases. These results may further improve the understanding of the structure–activity relationship for future GIPR-targeting PET tracers and further development of analogs with improved circulatory half-life and tissue exposure, as well as with higher binding affinity while preserving low liver background uptake.

## Figures and Tables

**Table 1:** Sequence alignment of native GIP(1-42) ligand, exendin-4, C803-GIP, and various other analogs, which previously have been used for GIPR imaging studies. C803-GIP is based on the hGIP(1-30) sequence and incorporates eight amino acid residue substitutions unique to C803-GIP. It is also amended with nine more amino acid residues, seven of which correspond to the exendin-4 sequence. The residues conserved from hGIP(1-30) are highlighted in yellow, the residues conserved from exendin-4 are highlighted in orange, the residues conserved from both hGIP(1-30) and exendin-4 are highlighted in blue, and the residues unique to C803-GIP are not highlighted.

	1	2	3	4	5	6	7	8	9	10	11	12	13	14	15	16	17	18	19	20	21	22	23	24	25	26	27	28	29	30	31	32	33	34	35	36	37	38	39	40	41	42
1	Y	A	E	G	T	F	I	S	D	Y	S	I	A	M	D	K	I	H	Q	Q	D	F	V	N	W	L	L	A	Q	K	G	K	K	N	D	W	K	H	N	I	T	Q
2	Y	A	E	G	T	F	I	S	D	Y	S	I	A	M	D	K	I	H	Q	Q	D	F	V	N	W	L	L	A	Q	K												
3	Y	a	E	G	T	F	I	S	D	Y	S	I	A	L	D	K	I	H	Q	Q	E	F	I	E	W	L	K	A	G	G	H	P	S	G	A	P	P	P	S*			
4	Y	Aib	E	G	T	F	I	S	D	L	S	I	A	L	D	R	I	H	Q	E	E	F	I	E	W	L	L	A	G	G	K *											
5	Y	A	E	G	T	F	I	S	D	Y	S	I	A	M	D	K *	I	H	Q	Q	D	F	V	N	W	L	L	A	Q	K												
6	Y	A	E	G	T	F	I	S	D	Y	S	I	A	Nle	D	K	I	H	Q	Q	D	F	V	N	W	L	L	A	Q	K *												
7	Y	A	E	G	T	F	I	S	D	Y	S	I	A	M	D	K	I	H	Q	Q	D	F	V	N	W	L	L	A	Q	K	G	K	K	N	D	W	K **	H	N	I	T	Q
8	H	G	E	G	T	F	T	S	D	L	S	K	Q	M	E	E	E	A	V	R	L	F	I	E	W	L	K	N	G	G	P	S	S	G	A	P	P	P	S			

1: hGIP(1-42); 2: hGIP(1-30); 3: C803-GIP; 4: S02-GIP-T4 (GIP1233); 5: [Lys16(Ahx-DOTA)]GIP(1– 30)NH2 (EG2); 6: [Nle14, Lys30(Ahx-DOTA)]GIP(1–30)NH2 (EG4); 7: [Lys37(DTPA)]N-acetyl-GIP1-42); 8: exendin-4; * Functionalized with a DOTA chelator moiety via cysteine amide; ** Functionalized with a DTPA chelator moiety.

**Table 2:** The potency of the half-maximal effective concentration (EC_50_, [pM]) for C803-GIP, Ga-C803-GIP, S02-GIP-T4, and Ga-S02-GIP-T4 measured in HEK293 cells transfected with the GIPR, GLP1-R, and GCGR.

	Human			NHP			Rat		
	GIPR	GLP-1R	GCGR	GIPR	GLP-1R	GCGR	GIPR	GLP-1R	GCGR
**C803-GIP**	0.7	704,000	999,999	1.1	181,000	999,999	12.7	381,000	999,999
**Ga-C803-GIP**	0.8	35,700	63,500	0.7	31,600	120,000	14.8	45,000	692,000
S02-GIP-T4 *	1.13	>10,000	>10,000	2.65	>10,000	>10,000	4.37	>10,000	>10,000
GaS02-GIP-T4 *	1.09	>10,000	>10,000	1.94	>10,000	>10,000	2.63	>10,000	>10,000

* [7].

**Table 3:** Estimated organ equivalent doses (mSv/MBq) of [^68^Ga]Ga-C803-GIP in human females and males extrapolated from the rat organ distribution data.

	Equivalent Dose (mSv/MBq)	
Tissue	Male	Female
Adrenals	0.00936	0.0124
Brain	0.000541	0.000658
Breasts	0.000934	0.00113
Gallbladder Wall	0.00515	0.00555
LLI Wall	0.00166	0.00196
Small Intestine	0.0031	0.00398
Stomach Wall	0.00335	0.00374
ULI Wall	0.00309	0.00371
Heart Wall	0.0319	0.0362
Kidneys	0.713	0.773
Liver	0.006	0.00754
Lungs	0.00576	0.0073
Muscle	0.00227	0.00306
Pancreas	0.00777	0.0089
Red Marrow	0.014	0.0135
Osteogenic Cells	0.0109	0.0145
Skin	0.000859	0.001
Spleen	0.0101	0.0124
Testes/Ovaries	0.00137	0.00189
Thymus	0.00178	0.00198
Thyroid	0.000487	0.000543
Urinary Bladder Wall	0.00152	0.00202
Effective dose (mSv/MBq)	0.0217	0.0237

## Data Availability

Data is contained within the article and supplementary material.

## Supplementary Materials

The following supporting information can be downloaded at: https://www.mdpi.com/article/10.3390/ph16010061/s1, Figure S1: Typical HPLC-chromatograms with UV absorption profile (lower panel) and radioactivity signal profile (upper panel) of [^68^Ga]Ga-C803-GIP; Figure S2: Polyacrylamide electrophoresis of the plasma incubated samples of [^68^Ga]Ga-GIP-C803. The left panel presents autoradiography image of the gel and the right panel presents protein image. Lane 1: Molecular weight Ladder; lane 2: [^68^Ga]Ga-GIP-C803 as a reference; lane 3: GIP-C803 (10 µM) as a reference; lane 4: [^68^Ga]Ga-GIP-C803 incubated in human plasma for under 30 s; lane 5: GIP-C803 (25 µM) as a reference; lane 6: [^68^Ga]Ga-GIP-C803 incubated in human plasma for 5 min; lane 7: GIP-C803 (50 µM) as a reference; lane 8: [^68^Ga]Ga-GIP-C803 incubated in human plasma for 45 min; lane 9: Human plasma sample; lane 10: [^68^Ga]Ga-GIP-C803 incubated in human plasma for 90 min; lane 11: Molecular weight Ladder; lane 12: [^68^Ga]Ga-EDTA as a reference for low molecular weight species; Figure S3. Representative images of the whole body distribution [^68^Ga]Ga-C803-GIP in a mouse carrying a GIPR expressing xenograft tumor. (A) A coronal projection over the plane of the kidneys and the xenograft tumor (white arrows). (B) Maximum Intensity Projection showing the three dimensionsional biodistribution. The colour scale is normalized to SUV = 10 and thus mainly the excretionary tissues kidney and bladder is visible. Figure S4. Time of flight mass spectrum of GIP-C803 with calculated monoisotopic mass of 4822.3 Da.
