# Supplementary material for: Imaging of the Glucose-Dependent Insulinotropic Polypeptide Receptor Using a Novel Radiolabeled Peptide Rationally Designed Based on Endogenous GIP and Synthetic Exendin-4 Sequences
Source: Pharmaceuticals (Basel). 2022 Dec 31;16(1):61. doi: 10.3390/ph16010061 (PMC9864903; doi:10.3390/ph16010061)
Supplement: Supplementary file 1 [file pharmaceuticals-16-00061-s001.zip › pharmaceuticals-2082441-supplementary.pdf]

Supplementary Materials

Imaging of the glucose-dependent insulinotropic polypeptide receptor using a novel radiolabeled peptide rationally designed based on endogenous GIP and synthetic Exendin-4 sequences

Irina Velikyan[1,2*], Martin Bossart[3*], Torsten Haack[3], Iina Laitinen[4], Sergio Estrada[1], Lars Johansson[5], Stefan Pierrou[5], Michael Wagner[3], Olof Eriksson[1,5]

1. Science for Life Laboratory, Department of Medicinal Chemistry, Uppsala University, Uppsala, Sweden
2. PET Centre, Centre for medical imaging, Uppsala University Hospital, Uppsala, Sweden
3. R&D Research Platform, Integrated Drug Discovery, Sanofi, Frankfurt, Germany
4. Global Imaging, Sanofi, Frankfurt, Germany; current address: Antaros Medical AB, Uppsala, Sweden
5. Antaros Medical AB, Mölndal, Sweden

*Corresponding authors:

Irina Velikyan
PET Center, Center for Medical Imaging
Uppsala University Hospital
SE-751 85 Uppsala, Sweden
Tel. +46 (0)70 4834137
E-mail: irina.velikyan@akademiska.se

Martin Bossart
R&D Research Platform
Integrated Drug Discovery
Sanofi
Frankfurt, Germany
E-mail: martin.bossart@sanofi.com

Radiolabeling of C803-GIP

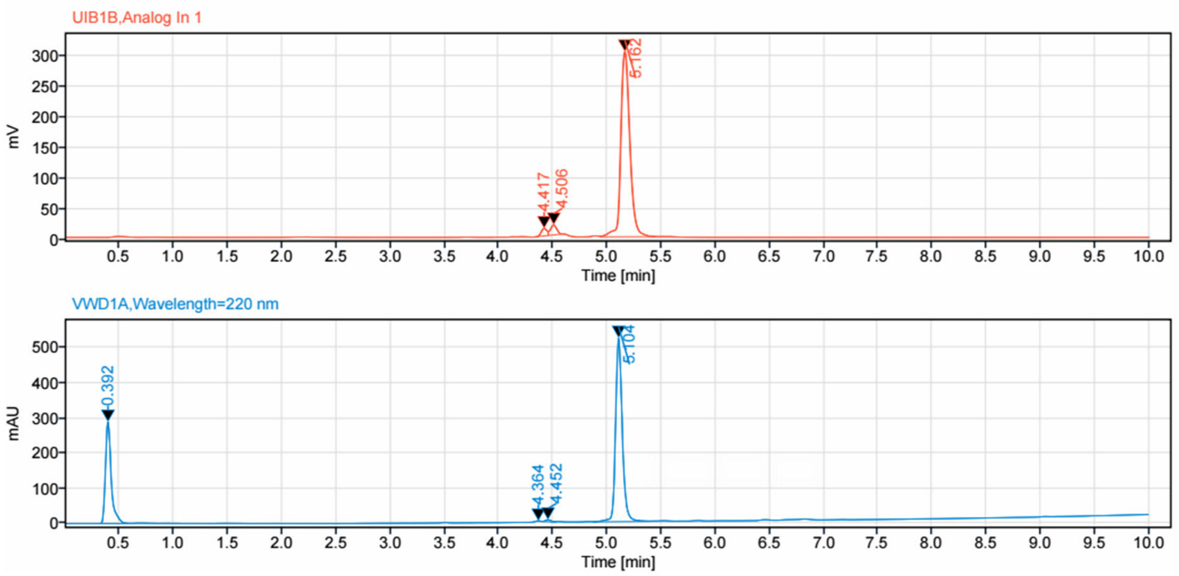

Figure 1S. Typical HPLC-chromatograms with UV absorption profile (lower panel) and radioactivity signal profile (upper panel) of [^{68}Ga]Ga-C803-GIP.

Stability of [^{68}Ga]Ga-GIP-C803 in human plasma

Stability of [^{68}Ga]Ga-GIP-C803 was studied in human plasma, wherein the agent was incubated at 37 ºC for under 30 seconds, 5 min, 45 min and 90 min. Thereafter, the samples were analyzed by polyacrylamide gel electrophoresis. To enable the interpretation of the results a number of references were applied along with the molecular weight marker (Figure 2S). The gel was imaged by GelDoc Go Imaging System (Biorad) for protein/peptide signals and thereafter exposed to a phosphor-imager screen overnight and digitalized using Cyclone Phosphor Imager system (PerkinElmer) for radioactivity signals. The amount of the sample radioactivity loaded on the gel was controlled by a reference drop of the same sample volume on a filter paper exposed to the same screen. The images were analyzed by ImageJ (NIH, US) and Image Lab (Biorad) software. [^{68}Ga]Ga-GIP-C803 was stable in human plasma for up to 90 min.

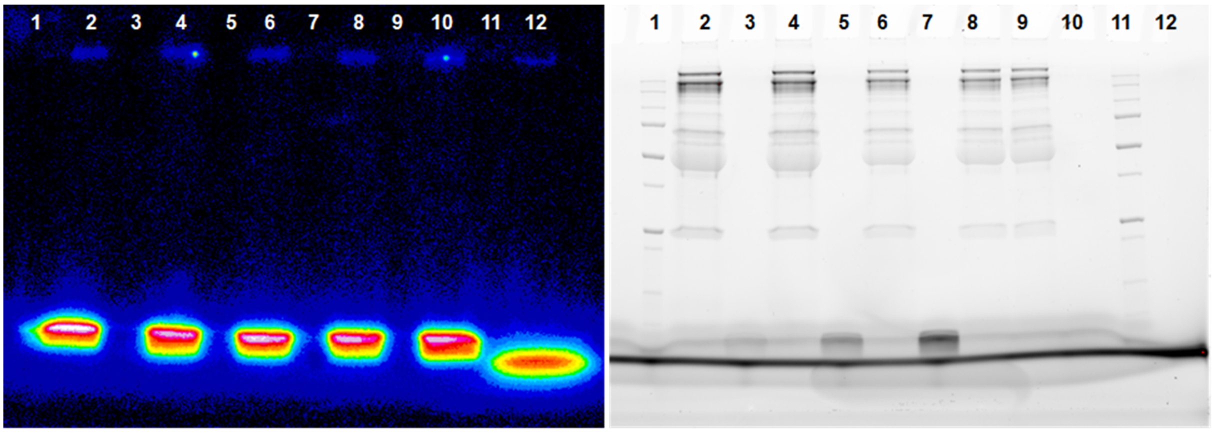

Figure 2S. Polyacrylamide electrophoresis of the plasma incubated samples of [^{68}Ga]Ga-GIP-C803. The left panel presents autoradiography image of the gel and the right panel presents protein image. Lane 1: Molecular weight Ladder; lane 2: [^{68}Ga]Ga-GIP-C803 as a reference; lane 3: GIP-C803 (10 µM) as a reference; lane 4: [^{68}Ga]Ga-GIP-C803 incubated in human plasma for under 30 sec; lane 5: GIP-C803 (25 µM) as a reference; lane 6: [^{68}Ga]Ga-GIP-C803 incubated in human plasma for 5 min; lane 7: GIP-C803 (50 µM) as a reference; lane 8: [^{68}Ga]Ga-GIP-C803 incubated in human plasma for 45 min; lane 9: Human plasma sample; lane 10: [^{68}Ga]Ga-GIP-C803 incubated in human plasma for 90 min; lane 11: Molecular weight Ladder; lane 12: [^{68}Ga]Ga-EDTA as a reference for low molecular weight species.

In vivo organ distribution of [^{68}Ga]Ga-GIP-C803 in mice

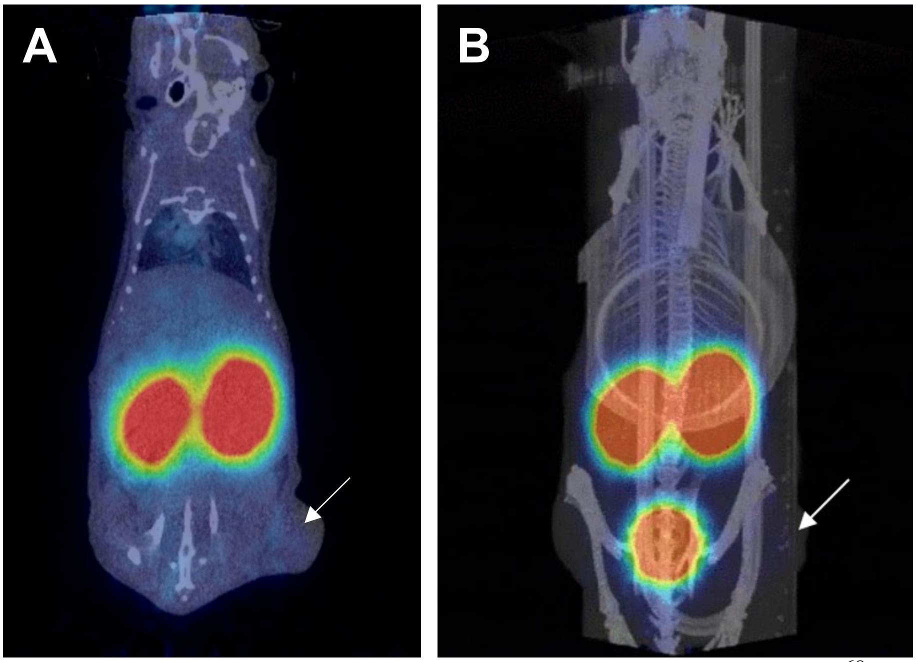

Figure 3S. Representative images of the whole body distribution [^{68}Ga]Ga-C803-GIP in a mouse carrying a GIPR expressing xenograft tumor. (A) A coronal projection over the plane of the kidneys and the xenograft tumor (white arrows). (B) Maximum Intensity Projection showing the three dimensionsional biodistribution. The colour scale is normalized to SUV=10 and thus mainly the excretory organs, kidney and bladder are visible.

Mass-spectrum of GIP-C803

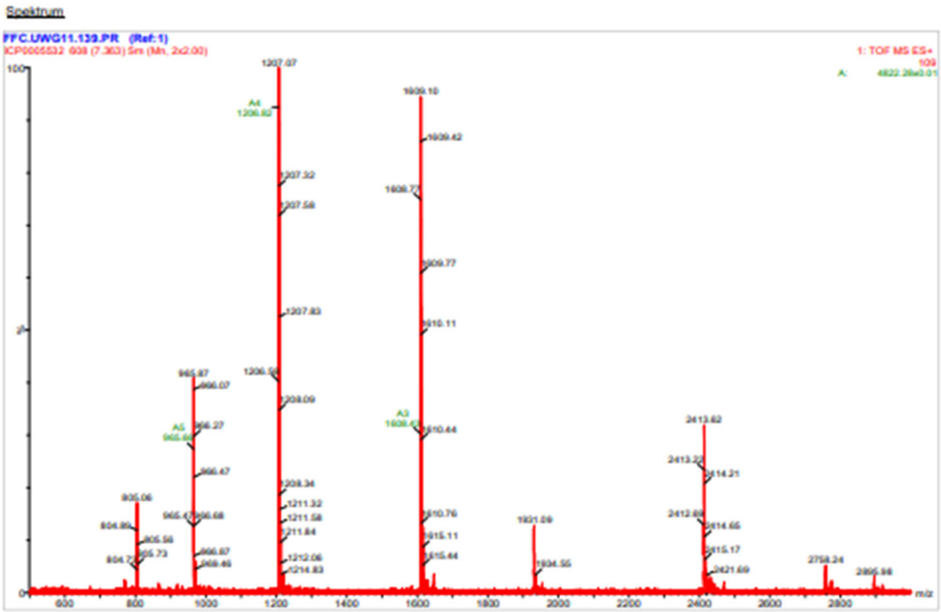

Figure 4S. Time of flight mass spectrum of GIP-C803 with calculated monoisotopic mass of 4822.3 Da.
